# Current and future applications of artificial intelligence in surgery: implications for clinical practice and research

**DOI:** 10.3389/fsurg.2024.1393898

**Published:** 2024-05-09

**Authors:** Miranda X. Morris, Davide Fiocco, Tommaso Caneva, Paris Yiapanis, Dennis P. Orgill

**Affiliations:** ^1^ Duke University School of Medicine, Duke University Hospital, Durham, NC, United States; ^2^ Department of Artificial Intelligence, Frontiers Media SA, Lausanne, Switzerland; ^3^ Harvard Medical School, Brigham and Women’s Hospital, Boston, MA, United States

**Keywords:** artificial intelligence, surgery, data science, machine learning, deep learning—artificial intelligence

## Abstract

Surgeons are skilled at making complex decisions over invasive procedures that can save lives and alleviate pain and avoid complications in patients. The knowledge to make these decisions is accumulated over years of schooling and practice. Their experience is in turn shared with others, also via peer-reviewed articles, which get published in larger and larger amounts every year. In this work, we review the literature related to the use of Artificial Intelligence (AI) in surgery. We focus on what is currently available and what is likely to come in the near future in both clinical care and research. We show that AI has the potential to be a key tool to elevate the effectiveness of training and decision-making in surgery and the discovery of relevant and valid scientific knowledge in the surgical domain. We also address concerns about AI technology, including the inability for users to interpret algorithms as well as incorrect predictions. A better understanding of AI will allow surgeons to use new tools wisely for the benefit of their patients.

## Introduction

Surgeons have the ethical duty to provide the best care to their patients in the setting of imperfect and constantly changing medical literature. The rapidly increasing volume and medical data collected by electronic health records has a growing need for computational resources to organize, interpret, and utilize the information (1). As these tools become more abundant, so too has the literature produced by the scientific community amidst this era of Artificial Intelligence (AI). AI is creating a technological revolution in society and is bound to affect the field of surgery (2).

**Figure 1:**
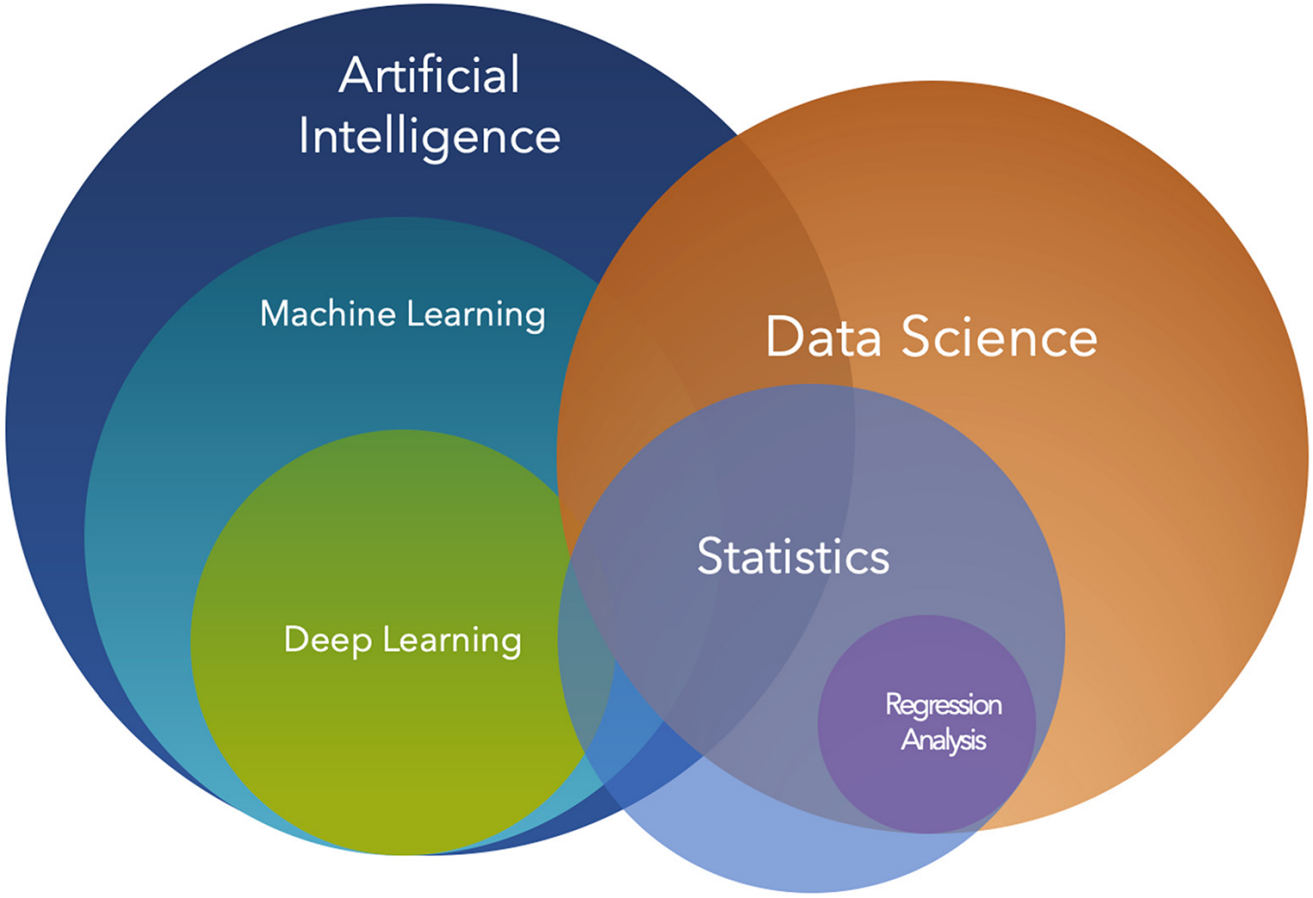
Venn diagram of artificial intelligence and data science. Artificial intelligence (AI) is a broad field that encompasses various sub-disciplines, including machine learning. Machine learning is a subset of AI focused on algorithms and statistical models that enable computers to perform tasks without explicit instructions, by relying on patterns and inference instead. Deep learning, a subset of machine learning, employs multi-layered neural networks to model complex patterns and decision-making processes. Data science, while related, is a distinct discipline that involves extracting knowledge and insights from structured and unstructured data. It heavily utilizes techniques from statistics and machine learning, among other methods. Within data science, regression analysis is a statistical technique used to understand relationships between variables and is often employed to predict outcomes.

In what follows, we define AI as the ability of a computer algorithm to perform tasks that typically require human intelligence. The roots of AI can be found in statistical learning and computer science. Traditional techniques in statistical learning such as regression analysis, which can be extended to non-linear and multivariate models, are useful to interpret surgical data and make predictions (Figure 1). Research in these fields has led to the development of increasingly complex and powerful machine learning models, culminating with the development of deep learning, which has revolutionized the field of AI by attaining advanced capabilities in tasks such as image recognition and natural language processing by combining the use of sophisticated algorithms, large datasets, and powerful computational resources. These innovations are bound to affect the field of surgery (2, 3).

Even more recently, the advent and broad availability of generative algorithms, such as AI chatbots and image generators, have raised more awareness about capabilities of AI that seemed almost impossible to attain just a few years ago (4). These systems, trained on large datasets, have the capability to correctly respond to user queries in natural language and generate realistic images. Furthermore, the technology has demonstrated high performance on medical content in the setting of standardized tests and graduate biomedical science exams (5). While potentially immensely useful, these systems have also raised concerns about harm they could cause, due to generation of incorrect output and “deepfakes” that can be difficult to detect.

To make the best recommendations for patients, surgeons rely heavily on the published literature and use what has been published as building blocks for future innovations. For innovation, there is a well-defined pathway between an idea and a product or procedure that is used by surgeons to treat patients. This includes basic science studies, pre-clinical studies, clinical studies and long-term outcome studies. In this article, we will focus on several of these steps and outline where AI is currently used, where there is potential, and concerns with use moving forward.

## AI in surgical education and training

The integration of AI in surgical education and training is an area of increasing interest and potential, with implications that extend across various domains of clinical practice and research.

### AI-driven personalized training modules

AI-driven personalized training modules in surgical education and training have emerged as a transformative approach, leveraging AI to provide individualized feedback and support to learners (6–8). This personalized approach enables access to a wide range of learning resources and offers insights to educators about how students are learning from their experiences (9). AI technology has the potential to optimize surgical practice, improve patient outcomes, and overcome barriers related to gender disparity in surgical training and education (10). Furthermore, the integration of AI in surgical education allows for the development of novel technology-enhanced learning platforms with personalized remote feedback, enhancing the overall learning experience (11). With the proper use of data, surgeons can achieve personalized decision-making for patients undergoing surgical procedures (12). Additionally, AI technology can be utilized to automate certain tasks of educators, personalize the learning experience, and improve learning outcomes in the field of education. Therefore, AI-driven personalized training modules hold significant promise in revolutionizing surgical education and training by providing tailored support and feedback to learners, ultimately enhancing the quality of surgical practice and patient care.

### Objective assessment of surgical skills using AI analytics

The use of AI in surgical education and training involves the utilization of AI algorithms to analyze data collected during surgical simulations or actual procedures. Guerrero et al. (13) discuss the use of AI in surgical education, emphasizing its role in providing customized adaptation, including performance assessment and feedback to surgical trainees (13). Cacciamani et al. (14) highlight the use of machine learning to identify and classify suturing gestures, create automated objective evaluation reports, and determine surgical technical skill levels to predict clinical outcomes (14). Soangra et al. (15) discuss the adoption of tools like Objective structured assessment of technical skills (OSATS) for graded evaluation based on specific criteria, such as respect for tissue, time and motion, instrument handling, flow in operation, and overall performance (15). Additionally, Singh et al. (16) emphasize the limitations of traditional evaluation methods and the potential of AI-based assessment tools to provide objective feedback, overcoming inter-observer bias and limited expert availability (16). These references collectively support the concept of using AI analytics to evaluate technical skills, decision-making, and overall proficiency in surgical education and training, providing actionable feedback to learners and educators.

### Advancements in surgical education curricula

Advancements in surgical education curricula have been significantly influenced by the integration of AI in training methods and assessment tools. AI has not only transformed training methods and assessment tools but is also driving advancements in educational curricula. The use of AI enables the development of adaptive learning programs that continuously evolve based on the latest surgical techniques, technologies, and best practices, ensuring that surgical education remains current and relevant. This is supported by Ward et al. (17), who emphasize the current applications of AI in research and its potential impacts on surgical education (17). Furthermore, AI in curricular development facilitates a data-driven approach to education, where training programs are constantly refined and optimized based on performance outcomes and learner feedback (18). Findings by Weidener and Fischer (19), suggest that medical curricula should move towards including the topic of AI in medicine to develop the knowledge, understanding, and confidence needed to use AI in the clinical context (19). The incorporation of AI in surgical education is also highlighted by Park et al. (8), who discuss the role of AI in surgical simulation, emphasizing the importance of fidelity in representing reality within surgical simulators (8). Additionally, the study by Moglia et al. (20) demonstrates the use of deep-learning models to predict proficiency acquisition in robot-assisted surgery, providing insights for personalized training within surgical programs (20). With respect to board exam preparation, Oleck, et al. discuss the implementation of ChatGPT as a tool to aid plastic and reconstructive surgery residents for oral board examinations (21). Therefore, the integration of AI in surgical education not only enhances the learning experience but also contributes to the overall improvement of surgical training and assessment.

The application of AI in surgical education and training is a burgeoning field that holds significant promise for enhancing the quality of surgical training, ensuring consistent and objective assessment of skills, and driving the continuous improvement of surgical education. As these technologies continue to evolve and integrate into surgical training programs, they are poised to play a pivotal role in shaping the future of surgical training, ultimately leading to improved patient outcomes and the advancement of the surgical profession. The ongoing collaboration between clinicians, educators, and AI experts will be crucial in realizing the full potential of these technologies, ensuring that they are leveraged effectively and ethically to enhance surgical training and patient care.

## AI in the publishing domain

AI can be useful to facilitate various steps of the evaluation, production and dissemination of scientific articles mediated by publishing houses (22). In the realm of scientific article review and evaluation, one significant challenge is efficiently managing a high volume of submissions, which results in increased publication output (23). This challenge gave rise to the utilization of AI as a tool for streamlining the process. By leveraging AI, publishing houses can aim to scale their operations while ensuring thorough scrutiny and maintaining the integrity of publications.

To address the issue, automated systems have been implemented (22). Right after an article is submitted, AI can facilitate the process of its thematic classification (24), e.g., identifying articles that are more apt for adjacent medical fields rather than surgical. Utilizing techniques like TF-IDF or more sophisticated document embeddings (25, 26), can allow reclassification ensuring that the research reaches its relevant audience.

AI offers an extra layer of quality control by highlighting areas that warrant the attention of peer reviewers. By doing so, AI arguably improves both the speed and quality of the review process (22). One example of this kind is the detection of image manipulation including copy-move forgery. Utilizing computer vision algorithms point descriptors it's possible to identify areas of similarity within e.g., microscopy images that are indicative of image manipulation (27). AI can be used to combat the surge of mass-produced content often released by paper mills (28). By using decision trees (29) harnessing classifiers and models like BERT (30), which are trained on historical data, it's possible to identify and weed out manufactured content also monitoring the integrity of the peer-review process (31).

AI-backed systems can scrutinize potential weaknesses in the research methodology in articles. Articles based on non-validated in-silico techniques (32), can be detected with the methods mentioned above in the context of text classification, trained on labeled data. AI is utilized by publishers to find reviewers who have the expertise to evaluate and provide feedback to authors during peer-review. For this purpose, a variety of approaches can be employed, and similar approaches can be applied to discover articles of interest for a given researcher (33, 34).

It is possible to harness the power of AI in early identification of high-impact articles, facilitating rapid dissemination of potentially ground-breaking research (35). This predictive intelligence could aid publishers or researchers and help medical practitioners in staying abreast with the most pivotal advancements in their field. However, we need to note that the use of AI should not compromise the essential human aspects of scientific publishing. Editors and peer reviewers continue to play critical roles in evaluating authenticity, contextual accuracy, and the unique perspective an article provides to its readers, especially in AI-generated research in surgery where the real-world implications of presented data are critical. Moreover, emphasis needs to be placed on informing stakeholders about the application and limitations of AI within operational procedures, ensuring transparency and trust in the system.

The Committee on Publication Ethics, or COPE, is working on the definition of industry-wide best practices and guidelines for AI-enhanced scientific publishing (36). By striking a balance between the capabilities of AI and the oversight of humans, publishing processes can protect the rigorous validity and quality of research publications. Consistent advancements in the field of artificial intelligence enable updates to the AI capabilities deployed in the scientific publishing process. This is not just to keep pace with emerging technologies but also to effectively counter the increasingly sophisticated maneuvers of malevolent actors, which may resort to breakthroughs in generative AI to produce fraudulent papers at scale (37).

## Intraoperative imaging with artificial intelligence

AI has been very helpful in diagnostic radiology in increasing the sensitivity and specificity of radiological examinations. Liu and colleagues have reviewed the development of AI to analyze CT scans and improve the predictive ability of the diagnosis of malignancy on pulmonary nodules (38). These successes of AI in radiology are now being translated into surgery. This has ushered in a transformative era, enhancing various facets of intraoperative procedures, and contributing significantly to the precision, efficacy, and efficiency of surgical interventions. AI has taken a pivotal role in intraoperative imaging, making considerable strides in image processing and analysis, real-time segmentation, and minimally invasive and robotic surgeries, supplemented by relevant case studies and clinical outcomes (39, 40).

### AI-enhanced image processing and analysis during surgery

The integration of AI algorithms in intraoperative image processing and analysis has led to a substantial enhancement in the quality and interpretability of surgical images. Advanced machine learning models, particularly deep learning, have demonstrated remarkable capabilities in noise reduction, contrast enhancement, and feature extraction, thereby facilitating a clearer visualization of anatomical structures and pathological entities. These improvements are instrumental in aiding surgeons to make more informed decisions and execute precise manipulations, ultimately contributing to improved surgical outcomes.

### Real-time image segmentation and augmentation for improved precision

AI-driven image segmentation has become a cornerstone in contemporary surgical practices, enabling the delineation of critical structures and regions of interest with unparalleled accuracy. Real-time processing capacities of AI ensure that these segmentations are promptly available, allowing for immediate utilization intraoperatively. Furthermore, image augmentation techniques, powered by AI, provide surgeons with enhanced visual cues and supplementary information, such as the demarcation of safe surgical margins or the highlighting of potential risk areas (41). ML models are increasingly utilized for surgical phase recognition, which is seen as a fundamental task in computer-assisted surgery, and a critical aspect for the effective implementation of machine learning into real-time operative applications (42). Phase recognition describes the identification of various steps and phases of a procedure. For instance, the identification of Calots’ triangle to achieve the critical view of safety is a surgical phase in a laparoscopic cholecystectomy. Automating the identification and labeling of surgical phases offers the potential to reduce operative errors and enhance surgical training.

### Applications in minimally invasive and robotic surgeries

The synergy of AI and intraoperative imaging has found substantial applications in the domain of minimally invasive and robotic-assisted surgeries. In these settings, the precision and consistency afforded by robotic systems are complemented by the image processing capabilities of AI, resulting in an overall enhancement of surgical performance. Machine vision, coupled with AI, enables robotic systems to interpret intraoperative imagery in real-time, ensuring accurate navigation, tissue identification, and execution of surgical tasks. This not only aids in minimizing surgical trauma but also expedites the recovery process, underscoring the transformative impact of AI in modern surgical practices. There have been numerous use cases in recent years within general surgery, with laparoscopic surgery comprising the majority. These include laparoscopic cholecystectomy, colectomy, and sleeve gastrectomy, with laparoscopic cholecystectomy as the most represented in the literature (42–48).

### Case studies and clinical outcomes

Empirical evidence, derived from a multitude of case studies and clinical trials, substantiates the benefits of AI in intraoperative imaging. Instances of AI applications in neurosurgery have demonstrated a marked improvement in tumor resection accuracy, with enhanced image guidance leading to better delineation between tumor and healthy tissue (49–55). Similar outcomes have been observed in orthopedic surgery, where AI-assisted image segmentation has played a crucial role in accurate implant placements (56). These clinical successes translate to tangible benefits such as reduced post-operative complications, shortened hospital stays, and improved patient prognoses, ultimately validating the integration of AI in surgical practices.

The incorporation of AI in intraoperative imaging is a testament to the relentless pursuit of precision and excellence in surgery. The enhanced image processing, real-time segmentation, and their applications in minimally invasive and robotic surgeries are poised to elevate the standards of surgical practice and pave the way for innovative research avenues, promising a future where the synergy of technology and surgery reaches new heights.

## Data visualization and analytics in surgery: a transformative AI application

### Utilizing AI for integrating and analyzing complex surgical datasets

**Figure 2:**
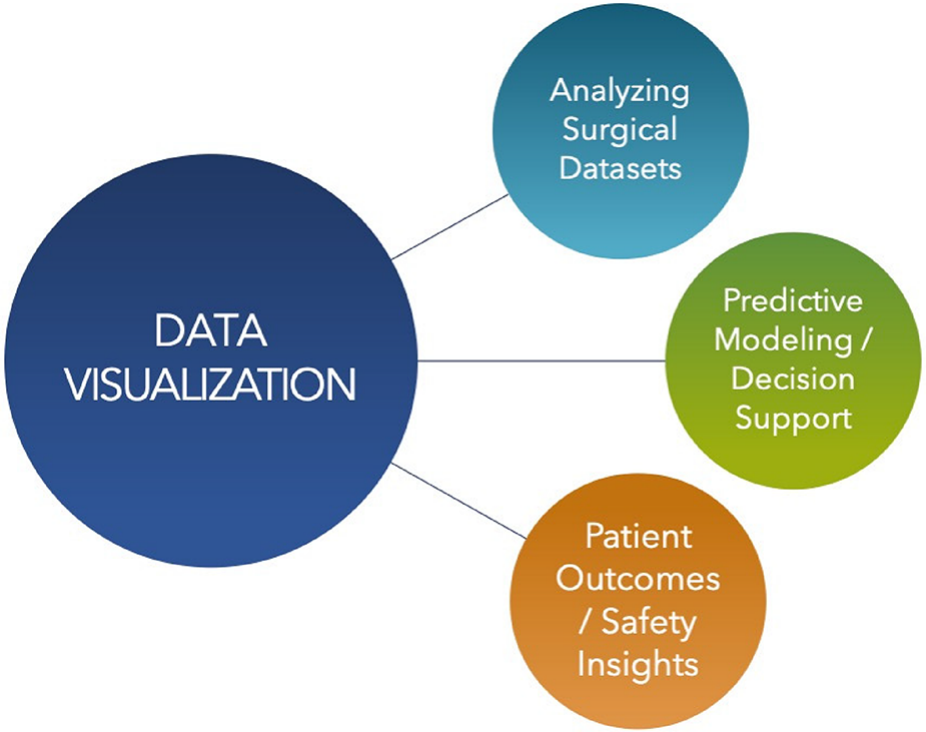
Visual summary of data visualizations and analytics. Usages of data visualization in surgery include analyzing surgical datasets, predictive modeling and decision support, and patient outcomes analysis, leading to safety insights.

**Figure 3:**
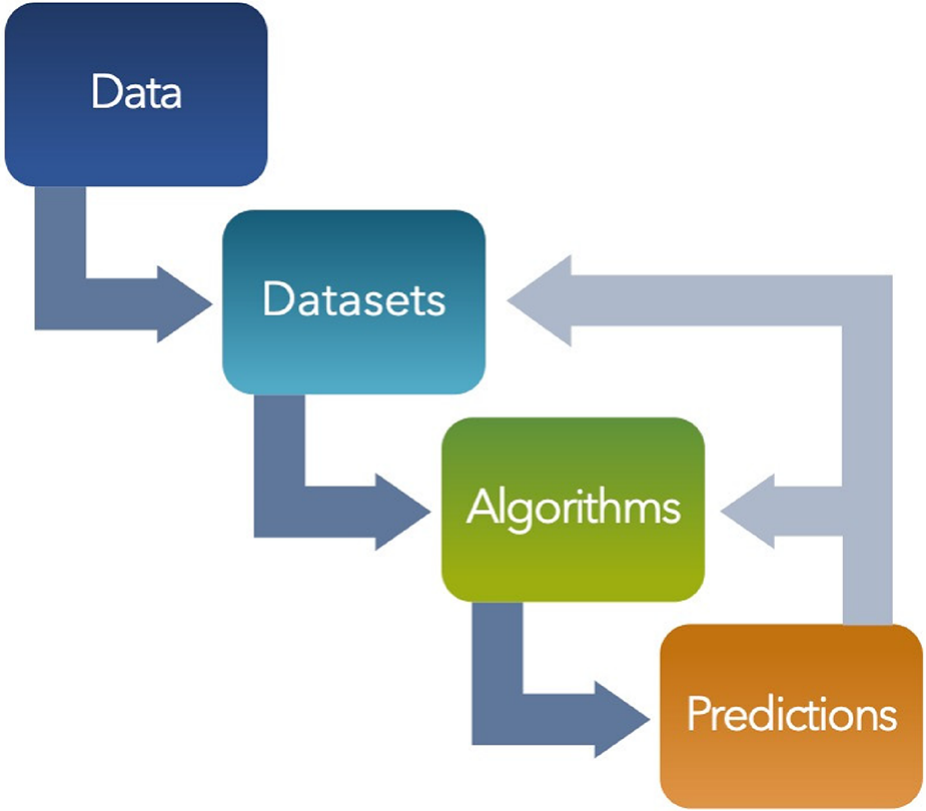
Visual diagram of flow and feedback loop from data to predictions. Predictive models can be monitored and continuously improved by tracking performance and validity. As new predictions are generated, they can be used to improve the performance of the existing algorithm or reveal underlying issues that may necessitate changes or additions to the original dataset. As models are built and deployed, performance metrics are collected to provide feedback to the model to for continuous improvement.

In surgery, vast amounts of data are available including pre-operative imaging, intra-operative monitoring, and post-operative care records. AI is proving invaluable in sifting through these datasets, facilitating their integration and analysis in a coherent manner. Machine learning algorithms, especially deep learning, have shown exceptional capability in identifying patterns and correlations within large, complex datasets, which are often beyond the scope of human analysis. By harnessing these capabilities, surgical teams can obtain a holistic view of patient data, leading to more informed decisions and personalized care strategies. A visual summary describing data visualizations and analytics is shown (Figure 2). The datasets compiled from the data are used to train algorithms, that are then utilized to generate predictions for various use cases. When implemented, AI algorithms generate predictions on new, unseen data which can be used to further fine-tune the algorithms for specific use cases. In addition, these predictions can be compared to historical values in the training and testing datasets to monitor for dataset drift, indicating that the algorithms may need to be retrained (57). If the collected metrics show poor performance that cannot be improved by modifying the algorithm it may indicate a need for expanding or diversifying the datasets used as well (Figure 3).

**Figure 4:**
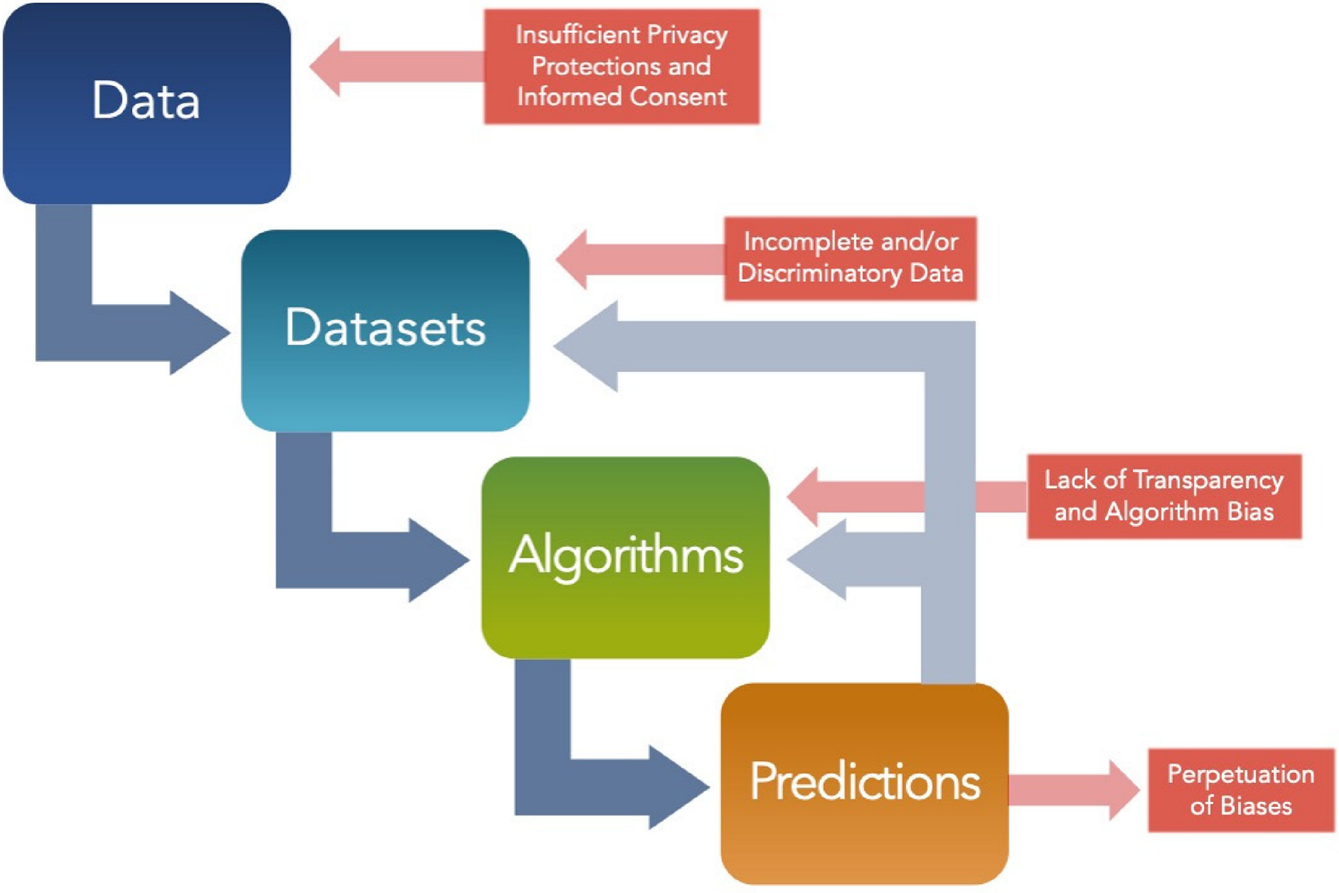
Ethical considerations throughout predictive modeling stages. Various opportunities for biases and other ethical considerations are depicted at multiple stages in the pathway from data to predictions. This includes but is not limited to insufficient privacy and inadequate informed consent within the data collection, incomplete and/or discriminatory data within the datasets, a lack of transparency and algorithmic biases, and finally perpetuation of biases that are reinforced by the model's predictions.

### Predictive modeling and decision support systems for personalized surgery

Predictive modeling and decision support systems play a crucial role in personalized surgery by forecasting potential complications and recommending optimal surgical approaches tailored to individual patient characteristics. These models enhance the precision of surgical interventions and reduce the risk of adverse outcomes (58). Decision support systems, powered by AI, act as invaluable tools for surgeons, providing evidence-based recommendations and augmenting the surgeon's expertise (59). For instance, in emergency surgery, artificial neural networks have been effective in predicting, diagnosing, and treating abdominal emergency conditions, such as acute appendicitis and acute cholecystitis (60). Additionally, a personalized approach to predicting the results of reconstructive surgery for chronic otitis media has been developed, aiding in choosing surgical tactics based on individual patient groups. Furthermore, the use of machine learning and mechanistic models has been proposed for personalized chemotherapy and surgery sequencing in breast cancer, incorporating tumor cell growth and the effects of chemotherapy and surgery under cell-kill hypotheses (61). These examples illustrate the potential of predictive modeling and decision support systems in revolutionizing personalized surgery, ultimately improving patient outcomes. The integration of such systems into the surgical workflow ensures a synergistic approach to patient care, where human judgment and AI-driven insights work in tandem.

### Data-driven insights for enhanced patient outcomes and safety

Data-driven insights play a crucial role in enhancing patient outcomes and safety in healthcare. AI systems have the potential to identify early signs of complications, allowing for timely interventions and improved recovery trajectories (62). For example, AI can analyze cardiovascular data to identify cancer patients at risk for cardiovascular complications early in treatment, enabling rapid intervention to prevent adverse outcomes (62). Additionally, the aggregation of anonymized surgical outcomes creates a rich database that enables the identification of best practices and benchmarks in surgical care (63). This approach allows for the customization of outcome reporting in surgery, improving reproducibility and comparability of data to ultimately enhance the quality of care (63). Furthermore, the use of benchmarking in surgical care has been shown to validate improved outcomes over time, despite increased complexity in procedures (64). By leveraging data-driven insights, healthcare providers can continuously improve surgical techniques and perioperative care, making surgery safer and expanding the eligibility of patients with more advanced diseases for surgical interventions (65).

## Perils and challenges of AI usage in surgery

The integration of AI and related technologies in the field of surgery raises significant ethical implications. These technologies have the potential to revolutionize surgical practices, from diagnostics to robotic surgery, but they also bring forth complex ethical concerns (Figure 4). The ethical issues encompass patient rights, data privacy, accountability for errors, technical robustness, privacy and data governance, transparency, diversity, non-discrimination, fairness, and the impact on human agency and empathy (66–68). Furthermore, the use of AI in surgery also raises concerns about bias and discrimination, transparency, and the need for informed consent (69). The complexity and breadth of these ethical issues necessitate careful consideration and proactive strategies to ensure the appropriate development and use of AI in surgery.

### Challenges and considerations in data privacy and security

Although AI brings tremendous potential benefits in transforming surgical data visualization and analytics, significant challenges remain, particularly in the realms of data privacy and security (68, 70). The sensitive nature of patient data necessitates stringent measures to protect against unauthorized access and potential breaches (70). Robust encryption methods, secure data storage solutions, and comprehensive data governance policies are needed to ensure patient confidentiality while leveraging AI for surgical innovation (70). Ethical considerations surrounding AI-driven decision-making in surgery must be diligently addressed, ensuring transparency, accountability, and the safeguarding of patient autonomy (71). The ethical implications of AI in surgery are crucial, and transparency is a critical ethical consideration in AI (72). Additionally, the use of AI in surgery requires careful consideration of the potential risks and long-term complications, as well as the ethical implications of decision-making processes (73). The development of AI has become increasingly mature and its intelligent decision-making has been applied to many aspects of human life, including medicine, necessitating a thorough understanding of the ethical risk factors and mechanisms involved (71).

To address these ethical implications, comprehensive guidelines and regulations will need to be generated to govern the use of AI in surgery. Guidelines should encompass considerations for privacy and security, transparency, responsibility, accountability, and informed consent (69). Additionally, there is a need for strategies to mitigate bias and discrimination in AI systems, ensuring fairness and non-discrimination in surgical practices (69). Efforts to enhance technical robustness and ensure the reliability of AI technologies in surgery are crucial to address ethical concerns related to patient safety and well-being.

Public perception and comprehension of AI and robotic surgery play a significant role in shaping the ethical landscape. Understanding the public's perspectives can inform the development of ethical frameworks and guidelines that align with societal values and expectations (74). Additionally, integrating ethics education into technical learning, even at the middle school level, can promote AI literacy and ethical awareness, addressing concerns related to the impact of AI on human society (75).

AI stands at the forefront of a paradigm shift in surgical data visualization and analytics, promising unprecedented levels of precision, personalization, and safety. While there are substantial challenges, especially in safeguarding patient data and ensuring ethical AI practices, the potential rewards in enhanced patient outcomes and operational efficiency are monumental. As we navigate this transformative era, continuous dialogue among clinicians, researchers, ethicists, and policymakers is paramount, ensuring that AI is harnessed responsibly and to its fullest potential in the realm of surgery. The ethical implications of AI and related technologies in surgery are multifaceted and require a comprehensive approach to ensure responsible development and use (Figure 4). By addressing concerns related to privacy, transparency, accountability, bias, and discrimination, and integrating ethical considerations into education and policymaking, the ethical challenges associated with the use of AI in surgery can be effectively managed.

### Societal implications

As other technological innovations, AI has the potential to disrupt major aspects of our economy.

Previous technological disruptions have resulted in job loss in “blue collar” workers whereas AI will likely cause disruptions across our entire economy including white collar workers.

According to the ILO, tasks associated to specialist medical practitioners have a low risk of being automated (76). Still, AI could take over or improve time consuming tasks such as documentation, billing and routine patient communication. In the best case, this would allow physicians to spend more time with their patients through providing procedures that improve health or counseling.

## AI applications for surgical outcomes prediction

There have been several studies that predict operative risks associated with surgery. The American College of Surgeons has a surgical risk calculator with a focus on major operations and risks of morbidity and mortality (https://riskcalculator.facs.org/RiskCalculator/). It is particularly helpful in large operations in patients with several co-morbid conditions. It is based on the NSQIP database, which derives 30-day risk-adjusted, post-operative data from over 700 large medical centers. Over 600 of these are in the US in 49 of 50 states and the remainder are in 11 additional countries. This is based on a regression analysis of their large database.

**Figure 5:**
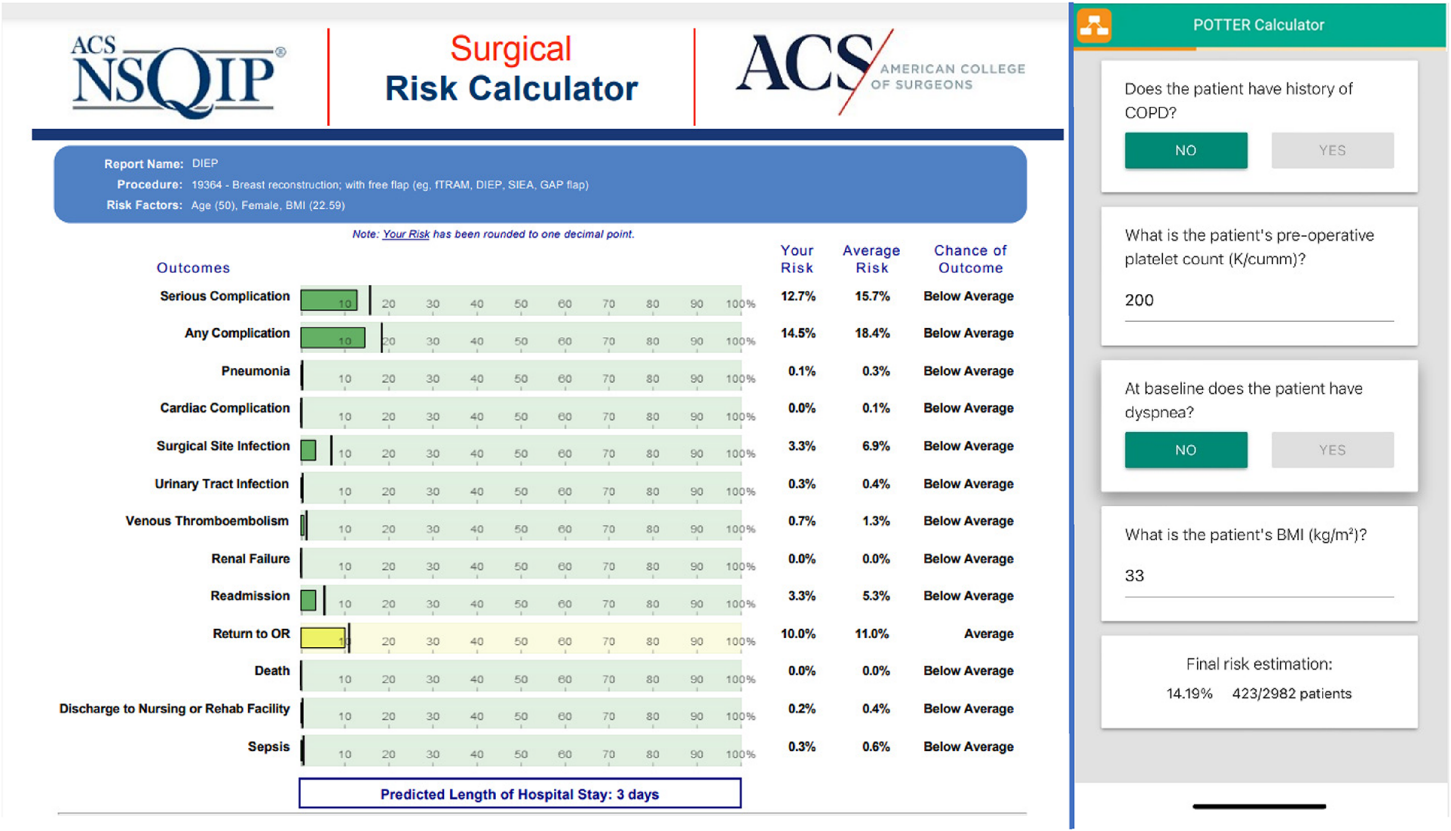
Examples of a regression analysis national surgical quality improvement program (NSQIP) surgical risk calculator, and AI app (POTTER). The left side depicts an example of the National Surgical Quality Improvement Program (NSQIP) risk calculator by the American College of Surgeons predicting complications following autologous breast reconstruction with free flap. Their extensive risk-adjusted, outcomes-based program is designed to measure and improve the quality of surgical care, by predicting data-driven outcomes based on patient characteristics and comorbidities. On the right is the risk of an emergency surgery of a 66-year-old patient with some medical co-morbidities (POTTER).

A more sophisticated artificial intelligence-based approach, POTTER, calculates the risk of Emergency Surgery and is available as a smartphone application (77, 78). These data-rich programs have helped specific hospitals focus and improve quality. As a result, for many of our cases, such as an autologous breast reconstruction with a free flap (Figure 5), both the risks of complications and mortality are low. For patients asking quality of life questions, better methods to assess these risks and convey them to the patient would be very helpful. For instance, future applications may help patients visualize how their breast reconstruction outcomes and scars may appear following a variety of reconstruction methods with AI-assisted visualization tools that show customized before-and-after images. Having this available directly to patients would better educate them and could augment a surgical consultation.

## Conclusion

AI technology heralds a transformative era characterized by enhanced precision, personalized care, and improved surgical outcomes. With its advanced data analytics and machine learning capabilities, AI facilitates processing of complex images, real-time decision-making, and predictive modeling, enabling surgeons to execute complex procedures with greater accuracy and confidence. It serves as an extremely valuable tool in preoperative planning, intraoperative guidance, and postoperative care, promising to elevate the standards of surgical practice. Moreover, AI's potential in surgical education and training is well-positioned to enrich learning experiences and objective skills assessment, fostering a new generation of adept surgeons. As the technology continues to mature, AI stands as a cornerstone of innovation, poised to reshape the future of surgery by offering smarter, safer, and more effective surgical interventions.

As AI evolves, its applications in surgery have shown significant promise in improving outcomes and patient safety. Surgeons can leverage AI to interpret complex data swiftly, paving the way for innovative solutions to intricate medical problems. Nonetheless, the reliance on data integrity is paramount, as AI's learning is inextricably tied to the quality of its input. The detection of fraudulent publications and data anomalies remains a critical concern, with current solutions falling short of the need for robust verification mechanisms. Ideally, AI would be able to reliably alert us to data integrity issues such as the detection of fraudulent publications, but the current literature suggests the current technical solutions are insufficient (79).

AI systems are complex, and it is not straightforward to interpret their behavior. This complexity necessitates a cautious approach to integrating AI insights into surgical decision-making, ensuring that AI complements rather than overrides the surgeon's expertise and clinical judgment.

As AI improves in fraud detection, malicious actors will increase their sophistication, in a sort of “arms race” (80). Historically, medicine and surgery have exhibited surprising discoveries that contradict the common narrative, for instance; Helicobacter pylori for peptic ulcer disease, the practice of bloodletting for sepsis, and surgical implants that are no longer used. Surgeons should ultimately approach new technology with a balance of open-mindedness and caution. The ethical landscape of AI in surgery is multifaceted, involving considerations of patient privacy, data security, informed consent, and the mitigation of biases. As AI tools become more integrated into surgical workflows, it is imperative to maintain a balance between embracing technological advancements and upholding the values of patient autonomy and ethical practice.

Looking ahead, AI's role in surgery is poised to grow, with ongoing research and development likely to yield more sophisticated and reliable applications. The surgical community, in partnership with AI researchers, must remain vigilant and proactive in addressing the challenges posed by these technologies. By fostering an environment of ethical awareness and continuous learning, the future of AI in surgery can be shaped into one that upholds the highest standards of patient care and fosters innovation while ensuring the ethical and judicious use of AI. In summary, AI's potential to revolutionize surgery is significant, yet its adoption must be tempered with a commitment to ethical practices, data integrity, and a continued respect for the surgical profession's humanistic aspects. It is within this balanced framework that AI will find its most meaningful and sustainable role in the surgical field.
